# Upregulation of GnT-IVa and Its Critical Roles in ATRA-Induced Differentiation of Acute Promyelocytic Leukemia Cells

**DOI:** 10.3390/biom16050756

**Published:** 2026-05-21

**Authors:** Siming Zhang, Tomoya Isaji, Meng Zheng, Yue Wang, Tiangui Wu, Tsukushi Saito, Yuhang Zhou, Tomohiko Fukuda, Shinichiro Takahashi, Jianguo Gu

**Affiliations:** 1 Division of Regulatory Glycobiology, Graduate School of Pharmaceutical Sciences, Tohoku Medical and Pharmaceutical University, 4-4-1 Komatsushima, Aoba-ku, Sendai 981-8558, Japan; 2 Department of Cancer Research Center, Nantong Tumor Hospital, The Affiliated Tumor Hospital of Nantong University, Nantong 226006, China; 3 Institute of Molecular Biomembrane and Glycobiology, Tohoku Medical and Pharmaceutical University, Sendai 981-8558, Japan; 4 Division of Laboratory Medicine, Faculty of Medicine, Tohoku Medical and Pharmaceutical University, 1-15-1 Fukumuro, Miyagino-ku, Sendai 983-8536, Japan

**Keywords:** GnT-IVa, N-glycan, cell differentiation, leukemia cells

## Abstract

Glycosylation is essential for hematopoietic cell homeostasis and malignant transformation. Dysregulated expression of glycosylation genes in leukemia cells accelerates disease progression and fosters drug resistance. Therefore, targeting these genes offers a promising avenue for anti-leukemic therapy. In this study, we explore the roles of N-glycans in acute promyelocytic leukemia (APL) differentiation using the ATRA-induced wild-type NB4 (WT/ATRA) or HL-60 cell model. We found that expression of N-acetylglucosaminyltransferase IVa (GnT-IVa, encoded by the *MGAT4A* gene) and its product (β1,4-GlcNAc-branched N-glycan) increased significantly during differentiation, as evaluated by lectin blot, real-time PCR, and flow cytometry. Interestingly, analysis of the Gene Expression Omnibus (GEO) public data showed that *MGAT4A* expression is significantly lower in APL patients, and higher *MGAT4A* expression was associated with favorable survival in AML cohorts. To address the role of GnT-IVa in differentiation, we established *MGAT4A*- and *MGAT4B*-knockout (KO) NB4 cell lines using CRISPR/Cas9. Compared to WT/ATRA cells, *MGAT4A* KO, but not *MGAT4B* KO, markedly suppressed ATRA-induced differentiation, as evidenced by reduced expression of *CD11b* and *CD11c*. We found that CD11b is a major glycoprotein carrying β1,4-GlcNAc-branched N-glycans. This modification enhanced CD11b stability, as CD11b expression declined more rapidly in *MGAT4A* KO cells in the presence of cycloheximide. In addition, *MGAT4A* KO suppressed ERK/MAPK signaling, which contributed to differentiation. Our study highlights the critical role of GnT-IVa in regulating APL differentiation, which may provide a basis for developing new differentiation therapies for APL.

## 1. Introduction

Acute promyelocytic leukemia (APL), a distinct subtype of acute myeloid leukemia (AML), is driven by the t(15; 17)(q22; q21) translocation, which produces the PML-RARα fusion oncoprotein [1]. This fusion protein functions as a transcriptional repressor of retinoic acid-responsive genes and arises from the arrest of differentiation at the promyelocytic stage [2,3,4]. Currently, the standard treatment for APL combines all-trans retinoic acid (ATRA) and arsenic trioxide (ATO), which has significantly improved patient outcomes. However, despite this success, differentiation-based strategies have not been successfully extended to the broader spectrum of AML, where outcomes remain suboptimal [5]. Therefore, elucidating the molecular mechanisms that govern leukemic cell differentiation remains an important goal to expand differentiation therapy beyond APL.

Differentiation arrest in APL patients involves a complex interplay of multiple mechanisms, including cell cycle arrest [6], metabolic reprogramming [7,8] and autophagy regulation [9]. Current evidence suggests that dysregulated genetic control alone cannot fully account for the complexity of differentiation arrest in APL. Instead, post-translational modifications—such as ubiquitination [10,11], sumoylation [12], and glycosylation [13,14]—are closely associated with differentiation treatment in APL patients. Among these, glycosylation is one of the most important protein modifications, playing essential roles in protein folding, conformation, localization, stability, and activity [15]. Notably, aberrant protein glycosylation has been strongly linked to tumor development and progression [16]. For instance, N-acetylglucosaminyltransferase III (GnT-III, encoded by the *MGAT3* gene) played a critical role in regulating erythroid differentiation in chronic myeloid leukemia [17]. Alpha2,3-sialyltransferase ST3GAL4 influenced the sensitivity of AML cells to Siglec-9-expressing macrophages, providing a possible target for immunotherapy in AML [18]. A combination of the fucosylation inhibitor 6-alkynylfucose (6AF) and ATRA significantly enhanced cell differentiation [13]. These studies highlight the significant roles of glycosylation in leukemia treatments. Additionally, some glycoproteins, such as CD79, CD82, and CD110, in leukemia cells have been reported to display abnormal glycans [19,20,21,22]. Despite advances in the field, the mechanisms by which N-glycans regulate differentiation in APL patients remain poorly understood, underscoring the need for further investigation. The NB4 and HL-60 cell lines, derived from APL and AML, respectively, provide a well-established model for studying ATRA-induced differentiation and offer a valuable system for identifying key regulatory pathways.

In this study, we observed a marked upregulation of GnT-IVa and its product, β1,4-GlcNAc-branched N-glycans, during differentiation, and this upregulation correlated positively with patient prognosis. *MGAT4A* KO impaired differentiation progression via the ERK pathway, accompanied by reduced expression of the markers *CD11b* and *CD11c*. Meanwhile, treatment with U0126, a specific MEK inhibitor, significantly suppressed differentiation and the expression of β1,4-GlcNAc-branched N-glycans. These findings underscore the importance of GnT-IVa in APL differentiation. The induction of *MGAT4A* suggests potential strategies to enhance the efficacy of differentiation therapy, offering new avenues for therapeutic intervention.

## 2. Materials and Methods

### 2.1. Reagents and Antibodies

The following antibodies and reagents were used in the experiments: Antibodies against CD11b (ab133357) were purchased from Abcam, Cambridge, UK; PU.1 (2266), p44/42 MAPK (9102), phospho-p44/42 MAPK (Thr202/Tyr204) (4370), the peroxidase-conjugated secondary antibody against rabbit (7074S), and U0126 (9903) were purchased from Cell Signaling Technology, Danvers, MA, USA; biotinylated Phaseolus vulgaris erythroagglutinin (E4-PHA) (B-1385), biotinylated *Datura stramonium* agglutinin (DSA) (B-1185), biotinylated *Phaseolus vulgaris* leucoagglutinin (L4-PHA) (B-1115–2) and ABC kit (PK-4000) were from Vector Laboratories, Burlingame, CA, USA; biotinylated *Lens culinaris* agglutinin (LCA) (J207) was obtained from J-Oil Mills, Tokyo, Japan; the anti-GAPDH (G9545) antibody was acquired from Sigma; the PE-conjugated mouse anti-human CD11b antibody was from BioLegend, San Diego, CA, USA; PNGase F was from Roche, Diagnostics, Mannheim, Germany. Cycloheximide (CHX) (037-20991) was from Wako, Saitama, Japan.

### 2.2. Cell Culture and Morphological Analysis

The NB4 and HL-60 cells were obtained from the Cell Resource Center for Biomedical Research at Tohoku University, Sendai, Japan. Cells were cultured in RPMI 1640 (Wako, Saitama, Japan) supplemented with 10% fetal bovine serum in a humidified atmosphere at 37 °C with 5% CO_2_. During erythroid differentiation, cells were treated with ATRA at 100 nM for 1, 3, and 5 days. Differentiated cells were collected and smeared onto glass slides for Wright–Giemsa staining. Differentiation was evaluated using a ZEISS LSM 900 confocal microscope (Carl Zeiss, Oberkochen, Germany).

### 2.3. Acquisition of the GEO Database for Bioinformatics Analysis

Gene expression datasets GSE71014 and GSE13159 were obtained from the Gene Expression Omnibus (GEO) database (https://www.ncbi.nlm.nih.gov/geo/) (accessed on 22 July 2025). The GSE13159 dataset was used to validate the expression of characteristic genes [23,24]. After excluding certain samples, the analysis included 73 normal bone marrow samples and 34 bone marrow samples from AML patients with the t(15; 17)(q22; q21) translocation. For prognostic analysis, the GSE71014 dataset, which includes RNA-seq and survival data from 104 AML patients, was used. Based on gene expression, patients were assigned to low- and high-expression groups. Survival differences between the two groups were assessed by Kaplan–Meier analysis and compared using the log-rank test with the “survival” (version 3.8-3) and “survminer” (version 0.5.0) by R packages (version 4.3.3) [25] Xiantao Academic (https://www.xiantaozi.com/) (accessed on 22 February 2026) is a powerful bioinformatics analysis tool that offers extensive functionalities for gene correlation analysis [26].

### 2.4. Western Blot and Lectin Blot Analysis

Cells were washed with ice-cold PBS and lysed on ice for 30 min in lysis buffer (20 mM Tris–HCl, pH 7.4, 150 mM NaCl, 1% Triton X-100) containing protease and phosphatase inhibitors (Nacalai Tesque, Kyoto, Japan). Lysates were centrifuged at 15,000× *g* for 10 min at 4 °C, and the supernatant was collected. Protein concentrations were determined using a BCA protein assay kit (Pierce Biotechnology, Rockford, IL, USA). For pull-down assays, equal amounts of protein were incubated with DSA-agarose overnight at 4 °C with rotation. The beads were washed twice with TBS, and bound proteins were analyzed by Western blotting.

For Western and lectin blotting, equal amounts of protein were resolved by SDS-PAGE and transferred to PVDF membranes (MilliporeSigma, Billerica, MA, USA). Membranes were blocked with 5% BSA (for lectin blot) or 5% nonfat milk (for Western blot) in TBS containing 0.05% Tween-20 (TBST) for 90 min at room temperature, then incubated with primary antibodies or biotinylated lectins overnight at 4 °C. After washing with TBST, membranes were incubated with appropriate horseradish peroxidase (HRP)-conjugated secondary antibodies or HRP-conjugated streptavidin. Signals were visualized using Immobilon Western Chemiluminescent HRP Substrate (MilliporeSigma, Billerica, MA, USA). Original western blot images can be found at Supplementary Figure S4.

### 2.5. Establishment of MGAT4A KO Cells

The Lenti-CRISPR v2 plasmid (#52961) was obtained from Addgene (Watertown, MA, USA). *MGAT4A* (5′-GTCAGGATAATAGCTTTCAG-3′) and *MGAT4B* (5′-GAAGGAGGACTCGGTCATCG-3′) KO cells were generated by targeting the human GnT-IVa and GnT-IVb genes. Lentiviral particles were produced by co-transfecting 293T cells with the Lenti-CRISPR v2 vector, and the packaging plasmids psPAX2 (5.5 μg), pMD2.G (2.4 μg), and TAX1 (0.6 μg) to enhance viral titers [27]. Viral supernatants were collected 48 h post-transfection and used to transduce NB4 cells. After transduction, cells were selected with puromycin (0.5 μg/mL) and plated in 96-well plates for single-cell clone isolation. Genomic DNA was extracted from the clones, and the targeted region was analyzed by PCR using the primers listed in Table 1.

### 2.6. RNA Extraction and Quantitative Real-Time PCR (qPCR) Analysis

RNAs were extracted using TRI Reagent (MilliporeSigma), and 1 μg of total RNA was reverse-transcribed into complementary DNA using PrimeScript RT reagent with a genomic DNA Eraser (Takara Bio, Kusatsu, Japan) according to the manufacturer’s instructions. Primer sequences are listed in Table 2. PCR products were diluted to 50 ng/μL and analyzed on a StepOnePlus (Applied Biosystems, Foster City, CA, USA). Real-time PCR analyses were performed using TB Green Premix Ex Taq II (Tli RNaseH Plus) (Takara Bio) as described previously [28].

### 2.7. Flow Cytometry

Cells (1 × 10^6^) were washed twice with ice-cold PBS and incubated with the CD11b antibody in magnetic-activated cell sorting buffer (PBS containing 0.1% BSA) for 1.5 h on ice, with gentle mixing every 15 min. After washing, the cells were resuspended in 800 μL of cell sorting buffer. An isotype-matched PE-conjugated mouse IgG antibody (BioLegend, San Diego, CA, USA) served as a negative control. Fluorescence intensity was measured on an Attune flow cytometer (Thermo Fisher Scientific, Waltham, MA, USA) and analyzed using FlowJo V10 software. A vertical line (cut-off line) was set based on the fluorescence intensity of CD11b for the negative control samples. Cells to the right of the vertical line were defined as positive. The positive cell ratio was calculated as: Positive cell ratio (%) = (Number of cells to the right of the vertical line/Total number of cells in the histogram) × 100%.

### 2.8. Statistical Analysis

All data are presented as mean ± SD from at least three independent experiments. Statistical analyses were performed using GraphPad Prism 8.0.1 (GraphPad Software, Inc., San Diego, CA, USA) with one-way ANOVA and Tukey’s post hoc test, two-way ANOVA and Tukey’s multiple-comparisons test, or an unpaired Student’s *t*-test, as appropriate. Survival curves were generated using the KM method and compared using the log-rank test. Significance levels were set as follows: ns (not significant) for *p* > 0.05; * *p* < 0.05; ** *p* < 0.01; and *** *p* < 0.001.

## 3. Results

### 3.1. ATRA-Induced Differentiation in NB4 Cells

**Figure 1:**
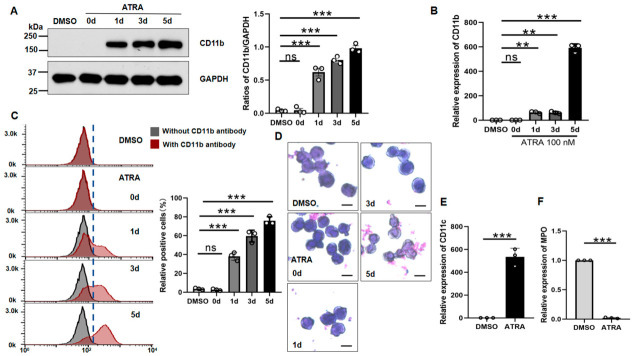
ATRA-induced differentiation of NB4 cells. (**A**) Western blot analysis of CD11b expression at the indicated time points. GAPDH served as a loading control. Band intensities were quantified and are presented as mean ± SD. (one-way ANOVA with Tukey’s test). (**B**) qPCR analysis of *CD11b* mRNA. Values were normalized to untreated wild-type (WT) cells (set as 1.0) and are shown as mean ± SD (*n* = 3). (one-way ANOVA). (**C**) Flow cytometric analysis of cell surface CD11b. The dotted line was set based on the fluorescence intensity of the negative control samples. Cells to the right of the vertical line were defined as positive. Quantified data are shown as mean ± SD (*n* = 3). (**D**) Cells treated with or without ATRA were stained with Wright–Giemsa stain. Scale bar: 10 μm. (**E**,**F**) qPCR analysis of *CD11c* (**E**) and *MPO* (**F**) mRNA. Normalization and data presentation as in (**B**). (unpaired Student’s *t* test). ** *p* < 0.01, *** *p* < 0.001, ns, not significant.

In this study, we induced differentiation of NB4 cells using ATRA, a well-established inducer. To determine the effect of ATRA on myeloid differentiation, NB4 cells were incubated with ATRA and then analyzed by Western blotting for CD11b, a myeloid monocyte differentiation marker. As shown in Figure 1A, ATRA significantly increased CD11b expression in a time-dependent manner. Quantitative PCR (qPCR) analysis revealed significant increases in *CD11b* mRNA levels following ATRA treatment (Figure 1B). Flow cytometry analysis confirmed significant upregulation of CD11b expression (Figure 1C). Consistent with these findings, morphological analysis of ATRA-treated NB4 cells showed a significant increase in differentiated cells, with nuclear indentation and bending, characteristic of granulocytic differentiation in metamyelocytes and the band stage. Furthermore, *CD11c* expression, another myeloid monocyte marker, was also increased during induction (Figure 1E). In contrast, myeloperoxidase (*MPO*) expression decreased during ATRA-induced differentiation (Figure 1F), consistent with previous reports [13,29]. These data suggest that ATRA at 100 nM effectively induces differentiation in NB4 cells over 5 days, as evidenced by changes in cell morphology and expression of differentiation markers.

### 3.2. The Expression Levels of β1,4-GlcNAc-Branched N-Glycans Increased in WT/ATRA Cells

**Figure 2:**
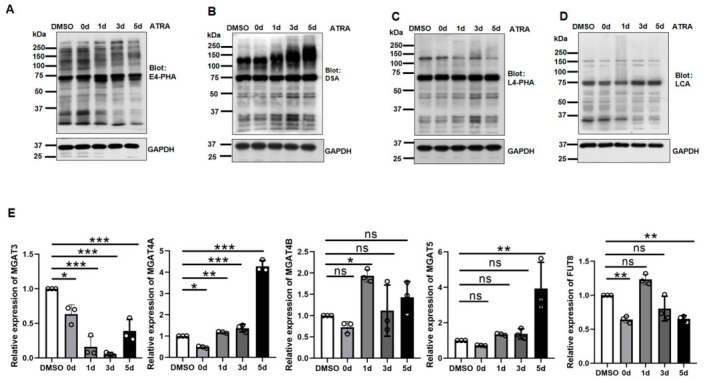
Comparison of N-glycan profiles and glycosyltransferase gene expression levels in WT and ATRA-treated NB4 cells. Cells were incubated with or without ATRA at the indicated times. Equal cell lysates were blotted with different lectins: (**A**) E4-PHA (bisected N-glycans), (**B**) DSA (β1,4-GlcNAc-branched), (**C**) L4-PHA (β1,6-GlcNAc-branched), (**D**) LCA (α1,6-fucose). GAPDH served as a loading control. (**E**) qPCR analysis of N-acetylglucosaminyltransferase genes involved in GlcNAc-branched N-glycan synthesis. GAPDH was used as an internal control, and values were normalized to the DMSO group (set as 1.0). Data are presented as mean ± SD. * *p* < 0.05, ** *p* < 0.01, *** *p* < 0.001; ns, not significant (one-way ANOVA with Tukey’s post hoc test).

To compare N-glycan expression patterns between WT and WT/ATRA cells, we performed lectin blot analysis using four distinct lectins: E4-PHA lectin preferentially recognizes bisected N-glycans, DSA lectin recognizes β1,4-GlcNAc-branched N-glycans, L4-PHA lectin recognizes β1,6-GlcNAc-branched N-glycans and LCA lectin recognizes α1,6-linked fucose. WT/ATRA cells showed a marked increase in DSA reactivity compared to WT cells, particularly after 5 days, coinciding with NB4 cell differentiation (Figure 2A). In contrast, little or no changes were observed in the other lectin blots, including E4-PHA (Figure 2B), L4-PHA (Figure 2C) and LCA (Figure 2D). qPCR analysis revealed that the mRNA levels of *MGAT4A*, but not *MGAT4B*, were significantly increased in WT/ATRA cells compared to WT cells (Figure 2E). The other genes, except *MGAT5*, showed no significant differences, supporting these lectin blot results.

We also examined HL-60, an AML-M2 cell line that can be induced to differentiate by ATRA. We analyzed CD11b expression in a time-dependent manner (Supplementary Figure S1A). Lectin blot analysis showed that ATRA induced a significant upregulation of glycans recognized by the DSA lectin during differentiation (Supplementary Figure S1B), suggesting that β1,4-GlcNAc-branched N-glycans are important for ATRA-induced differentiation.

**Figure 3:**
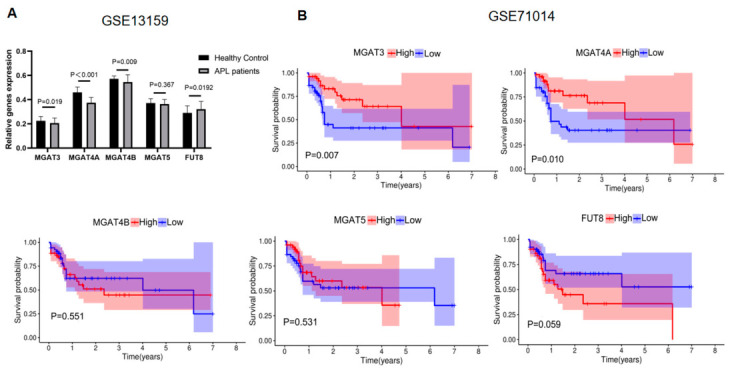
Validating expression levels of N-glycan-related genes in APL patients and their association with prognosis in AML cohorts. (**A**) Comparison of the expression levels of N-glycans-related genes between APL patients and healthy controls in the GSE13159 dataset. *p*-values were calculated using an unpaired Student’s *t*-test. (**B**) Comparison of survival rates between high and low expression of N-glycans-related genes in the GSE71014 dataset using Kaplan–Meier analysis.

Furthermore, we analyzed expression and its clinical value in two independent GEO datasets (GSE13159 and GSE71014). Remarkably, expression levels of N-glycan-regulated genes, except for *FUT8*, were significantly lower in APL patients than in normal subjects. Among them, *MGAT4A* (*p* < 0.001) showed the most significant differential expression (Figure 3A). Kaplan–Meier survival curves showed that higher expression of *MGAT4A* (*p* = 0.01) or MGAT3 (*p* = 0.007) was associated with better prognosis in AML cohorts. However, no significant difference in survival time was observed between the high- and low-expression groups for *MGAT4B*, *MGAT5*, and *FUT8* (Figure 3B). Therefore, *MGAT4A* may have prognostic relevance in AML.

### 3.3. MGAT4A KO, but Not MGAT4B KO, Suppressed ATRA-Induced Differentiation

**Figure 4:**
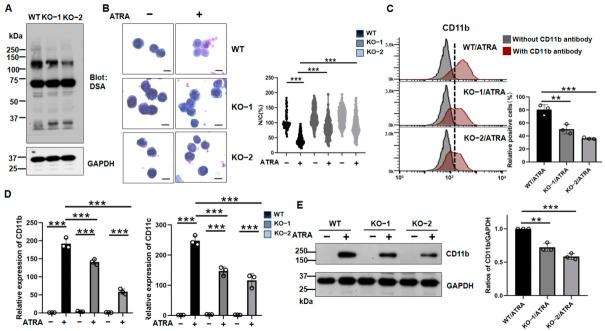
*MGAT4A* KO impaired ATRA-induced differentiation in NB4 cells. (**A**) Lectin blot analysis of WT and two independent *MGAT4A* KO clones, with or without ATRA treatment, using DSA. GAPDH served as a loading control. (**B**) Morphological assessment of WT and *MGAT4A* KO cells by Wright–Giemsa staining. Scale bar: 10 μm). One hundred cells in each sample were evaluated. Nucleus and cytoplasm areas were measured using ImageJ software (version 1.50i; National Institutes of Health, Bethesda, MD, USA), and the N/C ratio is shown as a violin plot. *** *p* < 0.001. (**C**) Flow cytometric analysis of cell surface CD11b expression. The dotted line was set based on the fluorescence intensity of the negative control samples. Cells to the right of the vertical line were defined as positive. Quantified data are shown as mean ± SD (*n* = 3). ** *p* < 0.01, *** *p* < 0.001. (**D**) qPCR analysis of *CD11b* and *CD11c* mRNA levels in WT and *MGAT4A* KO cells treated with or without ATRA. GAPDH was used as an internal control, and values were normalized to those in untreated WT cells (set to 1.0). Data are shown as mean ± SD from three independent experiments. *** *p* < 0.001. (**E**) Western blot analysis of CD11b expression in the indicated cell lines. GAPDH was used as a loading control. Band intensities were quantified and are presented as mean ± SD from three independent experiments. ** *p* < 0.01, *** *p* < 0.001.

To investigate the role of *MGAT4A*, we generated *MGAT4A* KO in NB4 cells using CRISPR/Cas9 and validated two single-cell clones by genomic sequencing (Supplementary Figure S2A). As expected, both *MGAT4A* KO clones showed reduced reactivity with DSA lectin compared with parental WT cells (Figure 4A). Compared with WT cells, *MGAT4A* KO cells showed markedly impaired differentiation in response to ATRA, as assessed by the nucleus/cytoplasm area (N/C) ratio using Wright-Giemsa staining (Figure 4B). We detected CD11b expression on the cell surface by flow cytometric analysis (Figure 4C). We next examined the expression of differentiation-associated markers. qPCR analysis revealed that ATRA-induced mRNA expression of *CD11b* and *CD11c* was significantly suppressed in *MGAT4A* KO cells compared with the WT cells (Figure 4D). Consistently, CD11b protein levels were also reduced in *MGAT4A* KO cells, as determined by immunoblotting of cell lysates (Figure 4E).

**Figure 5:**
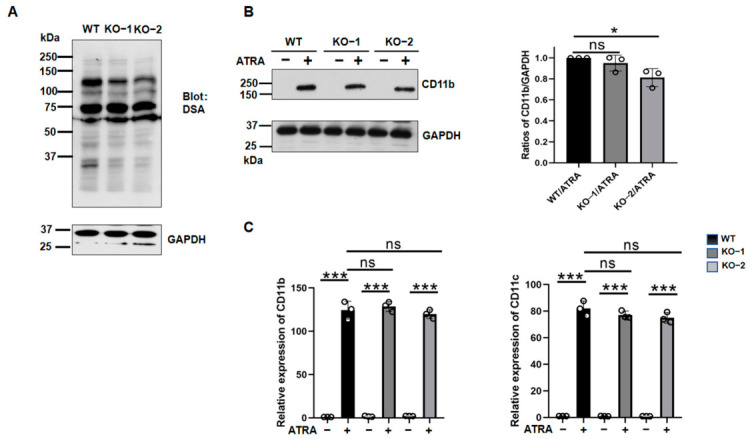
*MGAT4B* KO does not significantly impair ATRA-induced differentiation in NB4 cells. (**A**) Lectin blot analysis of WT and two independent *MGAT4B* KO clones, with or without ATRA treatment, using DSA. GAPDH served as a loading control. (**B**) Western blot analysis of *CD11b* expression in the indicated cell lines. GAPDH was used as a loading control. Band intensities were quantified and are presented as mean ± SD from three independent experiments. * *p* < 0.05; ns, not significant. (**C**) qPCR analysis of *CD11b* and *CD11c* mRNA levels in WT and *MGAT4B* KO cells treated with or without ATRA. GAPDH was used as an internal control, and values were normalized to those in untreated WT cells (set to 1.0). Data are shown as mean ± SD from three independent experiments. *** *p* < 0.001; ns, not significant.

To validate the effects of *MGAT4B* during ATRA-induced differentiation, we generated an *MGAT4B* knockout (KO) NB4 cell line using the CRISPR/Cas9 system and confirmed the knockout by genomic sequencing (Supplementary Figure S2B). The *MGAT4B* KO only slightly reduced DSA lectin reactivity, compared with WT cells (Figure 5A). Furthermore, the *MGAT4B* KO did not significantly suppress CD11b expression during ATRA-induced differentiation (Figure 5B). We then next examined the expression of differentiation-associated markers. As shown in Figure 4C, qPCR analysis revealed that ATRA-induced mRNA expression of *CD11b* and *CD11c* was not suppressed in *MGAT4B* KO cells compared with WT cells. These results strongly suggest that GnT-IVa and GnT-IVb may modify distinct substrates. Interestingly, Dr. Kizuka’s research group reported that GnT-IVa preferentially modifies larger glycoproteins, whereas GnT-IVb modifies proteins of approximately 75 kDa [30].

### 3.4. MGAT4A KO Suppressed PU.1 Expression and ERK Signaling During ATRA Induction

**Figure 6:**
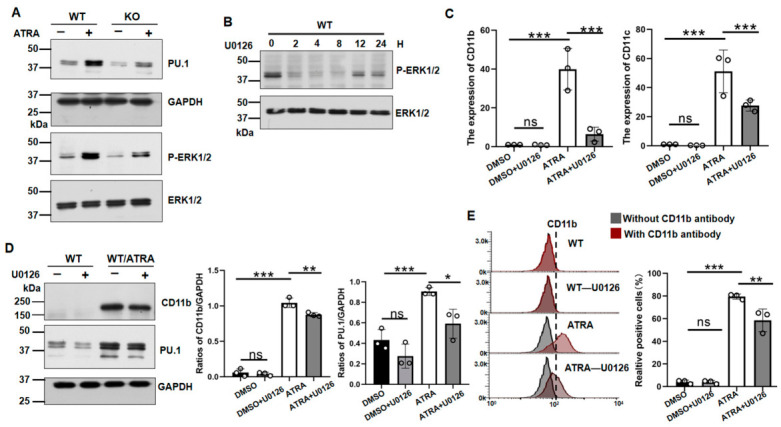
*MGAT4A* KO suppressed ATRA-induced differentiation partially through the MEK/ERK-mediated signaling pathway. (**A**) WT and MGAT4A KO NB4 cells were treated with or without ATRA, and the same amounts of cell lysates were Western blotted with anti-phosphorylated ERK1/2, total ERK1/2, and PU.1 antibodies. GAPDH served as a loading control. (**B**) Time-course analysis of ERK1/2 phosphorylation in WT NB4 cells treated with or without 1 μM U0126 for the indicated durations. (**C**) qPCR analysis of *CD11b* and *CD11c* mRNA levels in WT cells in the presence of ATRA treated with or without U0126. Values were normalized to GAPDH and are presented as mean ± SD (*n* = 3). *** *p* < 0.001; ns, not significant (one-way ANOVA with Tukey’s test). (**D**) Western blot analysis of CD11b and PU.1 expression. GAPDH was used as a loading control. Quantified data are shown as mean ± SD (*n* = 3). * *p* < 0.05; ** *p* < 0.01; *** *p* < 0.001; ns, not significant. (**E**) Flow cytometric analysis of cell surface CD11b expression. The dotted line was set based on the fluorescence intensity of the negative control samples. Cells to the right of the vertical line were defined as positive. Quantified data are shown as mean ± SD (*n* = 3). ** *p* < 0.01, *** *p* < 0.001; ns, not significant.

PU.1, encoded by the SPI1 gene, is a hematopoietic transcription factor that promotes myeloid differentiation and is upregulated upon ATRA treatment [31]. In addition, the ERK/MAPK cascade mediates ATRA-induced differentiation in APL cells, leading to increased PU.1 protein expression [32]. Here, we compared these changes between *MGAT4A* KO and WT cells. As shown in Figure 6A, PU.1 expression was markedly reduced in *MGAT4A* KO cells under ATRA induction compared with WT cells. ERK signaling activation was verified using Western blotting with an anti-phosphorylated ERK1/2 antibody. It clearly showed that the elevated levels of phosphorylated ERK1/2 in WT cells were neutralized in *MGAT4A* KO cells (Figure 6A). These results further support the involvement of *MGAT4A* in regulating ATRA-induced differentiation, which may be mediated by the ERK1/2 signaling pathway.

To confirm the role of the MEK/ERK signaling pathway in ATRA-induced differentiation, NB4 cells were treated with or without the MEK inhibitor U0126. U0126 effectively suppressed ERK1/2 phosphorylation within 1 h, and phosphorylation gradually recovered at 8, 12, and 24 h (Figure 6B), indicating that the inhibitory effect lasted approximately 8 h. To maintain sustained inhibition, the culture medium was replaced with fresh U0126 every 8 h throughout the induction period. The expression levels of *CD11b* and *CD11c* mRNA were significantly inhibited by U0126 (Figure 6C). In addition, inhibition was observed at the protein level by Western blotting with antibodies against CD11b and PU.1 (Figure 6D) and by flow cytometry (Figure 6E). Taken together, these results further confirmed that MGAT4A facilitates ATRA-induced differentiation, partially via the ERK/MAPK signaling pathway, positioning MGAT4A as a critical regulator of differentiation in APL cells.

### 3.5. Modification of CD11b by MGAT4A Prolonged Its Stability

**Figure 7:**
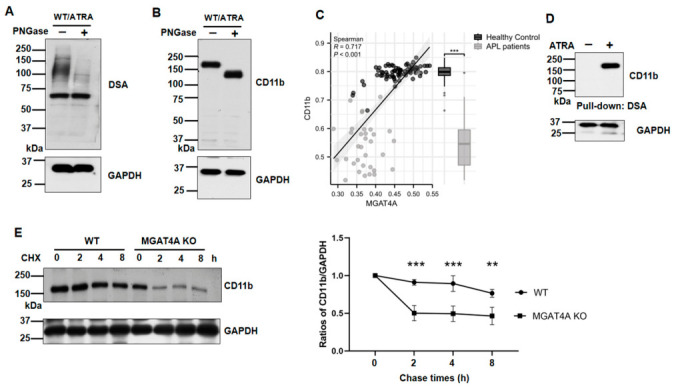
MGAT4A modified CD11b and increased its stability. Equal amounts of cell lysates were treated with or without PNGase F, and then either lectin-blotted with DSA (**A**) or Western-blotted with an anti-CD11b antibody (**B**). GAPDH served as a loading control. (**C**) Correlation between *MGAT4A* and *CD11b* expression and comparison between healthy controls and APL patients in the GSE13159 dataset. (**D**) A pull-down assay using DSA-agarose. Cell lysates from NB4 cells treated with or without ATRA were incubated with DSA-agarose, and the precipitates were immunoblotted with an anti-CD11b antibody. Input lysates were probed for GAPDH, which served as a loading control. (**E**) Protein stability assay. WT and MGAT4A KO cells were treated with 50 μM cycloheximide (CHX) for the indicated times. CD11b levels were assessed by Western blotting, with GAPDH as a loading control. Band intensities were quantified and normalized to their value at time 0, which was set to 1.0. Data are presented as mean ± SD from three independent experiments. ** *p* < 0.01, *** *p* < 0.001.

CD11b, also known as integrin alpha M, belongs to the integrin alpha chain family. Like other integrins [33], CD11b is a heavily N-glycosylated membrane protein [34,35]. PNGase F is an amidase that cleaves N-linked oligosaccharide chains from glycoproteins [36,37]. Following PNGase F treatment, reactivity with the DSA lectin was markedly reduced (Figure 7A). The molecular size of CD11b decreased, confirming that CD11b carries N-glycans (Figure 7B). Based on gene expression in the GSE13159 dataset, we observed a significant positive correlation between *MGAT4A* and *CD11b* levels (Spearman’s r = 0.717, *p* < 0.001), indicating that higher *MGAT4A* expression is associated with increased *CD11b* levels in healthy controls. In contrast, lower expression levels were observed in APL patients (Figure 7C). We observed no significantly positive correlation between *MGAT4B* and *CD11b* levels (Spearman’s r = 0.179, *p* = 0.066) in Supplement Figure S3A.

Then, we determined whether CD11b contains β1,4-GlcNAc-branched N-glycans, and a DSA pull-down assay was performed. As shown in Figure 7D, CD11b was detected in ATRA-induced cells, indicating that it carries β1,4-GlcNAc modification upon ATRA induction. Branched N-glycans are known to stabilize glycoproteins expressed on the cell surface [33]. To investigate the functions of CD11b modified by MGAT4A, we assessed CD11b stability in the presence of cycloheximide (CHX), a protein synthesis inhibitor. Interestingly, CD11b decayed significantly more rapidly in MGAT4A KO cells than in WT controls (Figure 7E). Given that CD11b mediates cell spreading and regulates intracellular signaling, including the ERK/MAPK signaling pathway [38,39], these findings suggest that the role of MGAT4A in ATRA induction may be mediated, at least in part, by modifying CD11b, thereby enhancing its cell surface expression and promoting ERK activation, which is important for cell differentiation.

## 4. Discussion

In this study, we investigated the roles of N-glycans in ATRA-induced differentiation and found the following: (1) GnT-IVa expression and its products, β1,4-GlcNAc N-glycans, were significantly upregulated in the differentiated cells; (2) *MGAT4A* KO, not *MGAT4B* KO, suppressed differentiation, suggesting substrate specificity for modification by GnT-IVa and GnT-IVb; and (3) GnT-IVa mediated β1,4-GlcNAc modification of CD11b enhancing its stability, which may partially modulate intracellular signaling to regulate cell differentiation. These findings demonstrate that GnT-IVa promotes differentiation, uncover a previously unrecognized role for GnT-IVa in differentiation.

**Figure 8:**
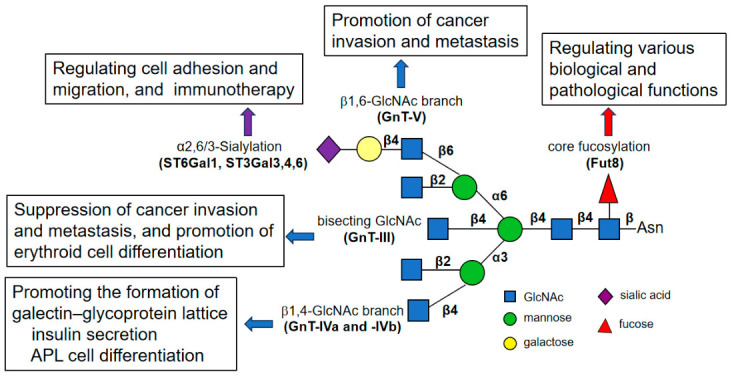
Roles of GlcNAc-branched N-glycans and others. Alterations in N-glycosylation patterns are common in tumors, and changes in glycan structures can influence various cellular behaviors. These include altered cell adhesion, migration, invasion, and differentiation, all of which affect tumor progression. In this study, we found that GnT-IVa expression promotes APL cell differentiation.

Many important questions remain about the role of GlcNAc-branched N-glycans. During N-glycan processing in the Golgi apparatus, the formation of a variable number of branches significantly increases the structural complexity of N-glycans [40]. As shown in Figure 8, each GlcNAc branch has distinct functions in the development and progression of various diseases by regulating the activities of specific glycoproteins [33]. For example, the β1,6 GlcNAc branch catalyzed by GnT-V significantly contributes to cancer invasion and metastasis [41,42]. In contrast, the β1,4 GlcNAc branch catalyzed by GnT-IVa plays a crucial role in regulating the glucose transporter 2 (GLUT2) and thereby influencing insulin secretion [43,44]. Conversely, the expression of bisecting GlcNAc catalyzed by GnT-III is often downregulated in cancers. The bisecting GlcNAc enzymatically sterically and/or conformationally interferes with the activities of GnT-IVs and GnT-V [45,46]. These observations may relate to galectin binding. Galectins cross-link glycoproteins, forming dynamic microdomains or lattices that control various mediators of cell adhesion, migration, proliferation, survival, and differentiation [47].

Galectins can be viewed as a code for repeating minimal binding units, N-acetyllactosamine (LacNAc), which can be extended with poly-LacNAc, fucose, sialic acid, and sulfate on the GlcNAc-branched N-glycans of glycoproteins, mainly modified by GnT-IVs and V. Multivalency is a key feature of galectin binding, allowing crosslinking of multiple targets and the formation of a galectin–glycoprotein lattice on the cell surface that influences receptor levels and functions. This galectin lattice has been linked to tumor progression through its effects on growth factor signaling [47] and on the stability of membrane glycoproteins such as GLUT2 and GLUT4 [43,48,49]. The phenomenon could also apply to CD11b in this study. As shown in Figure 7, the stability of CD11b was significantly downregulated in *MGAT4A* KO cells. Which galectins were involved in this event remains an important question for further study.

GnT-IVa and GnT-IVb are isozymes that catalyze the transfer of GlcNAc to α1,3-linked mannose in the core structure of N-glycans via a β1,4 linkage (Figure 8) [50,51,52]. Due to its high affinity for donor and acceptor substrates, GnT-IVa, but not GnT-IVb, is regarded as the primary enzyme for the formation of complex-type N-glycans [30]. In addition, the expression patterns of *MGAT4A* and *MGAT4B* are different in mammalian tissues. For example, *MGAT4A* expression was high in gastrointestinal tissues, whereas *MGAT4B* was ubiquitously expressed [43,51]. In pancreatic cancer, *MGAT4A* was downregulated due to the promoter methylation, while *MGAT4B* was overexpressed [53]. In choriocarcinoma, integrin β1 was modified by GnT-IVa to promote tumorigenicity [54,55]. In endometrial cancer, GnT-IVa/Galectin9-mediated modification of GLUT1 promoted glucose metabolism, supporting tumor proliferation and invasion [56]. These studies suggest that upregulation of GnT-IVa across various cancer types may promote tumor progression. These studies suggest that upregulation of GnT-IVa across various cancer types may promote tumor progression. In contrast, our findings demonstrate that GnT-IVa plays a differentiation-promoting role in APL cells, and higher *MGAT4A* expression is associated with a favorable prognosis (Figure 2). This apparent discrepancy may reflect the context-dependent functions of glycosylation. Because glycosyltransferases modify specific glycoprotein substrates available in a given cellular environment, the biological consequences of GnT-IVa activity are likely shaped by cell lineage, differentiation status, and signaling context. In epithelial tumors, GnT-IVa-mediated glycosylation may preferentially stabilize growth factor receptors or metabolic transporters that support tumor progression, as described above, whereas in leukemic cells, it may enhance the stability of differentiation-associated glycoproteins such as CD11b, thereby promoting differentiation signaling. These findings suggest that GnT-IVa may exert distinct biological effects depending on substrate availability and cellular context.

Our previous study showed that GnT-III, which increased E4-PHA staining, played a critical role in butylate-induced erythroid differentiation associated with the ERK/MAPK signaling pathway [17]. In ATRA-treated APL cells, we observed a decrease in E4-PHA staining alongside an increase in DSA staining; a similar observation has been reported previously [13], suggesting that distinct glycans play distinct roles in cell differentiation. This reciprocal change in N-glycans may also support the notion that GnT-III suppresses the formation of GlcNAc branches catalyzed by GnT-IV or GnT-V [57]. Thus, although GnT-IVa was the focus of this study, the functions of GnT-III in cell differentiation which could not be ruled out require further study. In addition, the ERK/MAPK signaling is required not only for butylate-induced erythroid differentiation [17] but also for ATRA-induced differentiation in APL cells [32] and this study.

PU.1 is a key hematopoietic transcription factor involved in myeloid, lymphoid, and erythroid differentiation and in regulating differentiation-associated genes such as CD11b [58,59]. Previous studies have shown that PU.1 is a downstream target gene of ATRA during APL treatment [60]. This study also showed that inhibition of ERK/MAPK signaling with the MEK-specific inhibitor U0126 suppressed ATRA-induced differentiation, PU.1 expression, and β1,4 GlcNAc-branched N-glycans, as assessed by DSA staining. Because PU.1 is a member of the Ets transcription factor family [61], these findings raise the possibility that Ets family transcription factors may participate in regulating *MGAT4A* expression during differentiation. In fact, Ets-1, another member of the Ets family, binds and trans-activates the *MGAT4A* and *MGAT5* promoters [62,63]. Together, these observations suggest a potential mechanistic link between Ets family transcription factors and GnT-IVa expression during ATRA-induced differentiation. On the other hand, we confirmed a positive regulatory relationship between *PU.1* and *CD11B* [64], and detected a positive correlation between *PU.1* and *MGAT4A* in GSE13159 (Supplementary Figure S3B,C).

It remains unclear why GnT-IVa regulates cell differentiation, but we could speculate that GnT-IVa modifies cell-surface glycoproteins, such as CD11b, thereby modulating intracellular signaling that regulates cell differentiation. CD11b, the integrin αM subunit, can bind to various ligands, including complement, fibrinogen, and ICAM-1. The diversity of ligand binding provides a structural basis for the functional diversity of CD11b. CD11b has been demonstrated to mediate the adhesion, migration, chemotaxis, and recruitment of macrophages during inflammation [65,66], and to regulate the phagocytic action of macrophages toward tumor cells [67], and to initiate intracellular signaling, such as ERK/MAPK signaling [38,39]. In fact, CD11b modified with β1,4-GlcNAc-branched N-glycans greatly enhanced its stability and cellular signaling (Figure 7). Furthermore, we found a positive correlation between *MGAT4A* and *CD11b* (Figure 7C), but *MGAT4B* was not significant (Supplement Figure S3A). This study revealed that MGAT4A may directly affect protein stability, such as that of CD11b, and also regulate the functions of certain membrane glycoproteins that participate in the ERK/MAPK signaling pathway, thereby indirectly affecting CD11b mRNA expression. The target proteins of GnT-IVa remain to be further studied, as discussed below. Although a previous study reporting GnT-IV expression is particularly interesting because enzymatic activity increases during oncogenesis and myelocytic cell differentiation by 1α,25-dihydroxyvitamine D3 and interleukin-6 (IL-6) [52], it should be noted that GnT-IVa, but not GnT-IVb, promoted ATRA-induced differentiation in this study. Thus far, we do not know the underlying mechanism. Dr. Kizuka’s research group reported that GnT-IVa and GnT-IVb may have preference acceptors for modification; GnT-IVa modifies the larger glycoproteins, whereas GnT-IVb modifies smaller proteins [30]. The precise mechanisms require further investigation.

Several limitations of the present study should be acknowledged. First, the findings were derived primarily from NB4 and HL-60 cellular models and were not validated in primary patient samples or in vivo leukemia models. Although these cell lines are well-established systems for studying ATRA-induced differentiation, they cannot fully recapitulate the genetic heterogeneity and microenvironmental complexity of APL in patients. Second, based on expression data from public databases, future studies involving larger cohorts with comprehensive clinical information and prospective patient sample collection will be necessary to further validate the clinical relevance of GnT-IVa in leukemia progression and differentiation therapy. Third, although DSA pull-down assays suggested the presence of β1,4-GlcNAc-branched N-glycans on CD11b, the precise N-glycan structures and site-specific glycosylation patterns were not directly characterized in this study. Comprehensive glycomics and glycoproteomics, using MALDI-TOF MS or LC-MS/MS, will provide more definitive structural information on CD11b and other glycoproteins, thereby clarifying the molecular mechanisms underlying GnT-IVa-mediated regulation of protein stability and differentiation signaling.

## 5. Conclusions

This study identifies GnT-IVa as a differentiation-associated glycosyltransferase that promotes ATRA-induced differentiation in APL cells. We demonstrate that GnT-IVa-mediated β1,4-GlcNAc branching is upregulated during differentiation and contributes to the stabilization of CD11b, thereby influencing ERK/MAPK signaling. These findings reveal a previously unrecognized role of GnT-IVa in leukemic differentiation and support the concept that glycosylation actively contributes to differentiation regulation rather than merely representing a secondary consequence of cellular maturation, highlighting glycosylation as a potential regulatory mechanism in APL.

## Figures and Tables

**Table 1:** Primer sequences for the target region.

Gene	Forward Primer	Reverse Primer
MGAT4A	TGGCTTAGGATTTCTATGCT	ATGTTTTCAGCTTCGATAAA
MGAT4B	GTCTTCCATCACCTGCCACA	AGCAACTGAACTTCCGACAG

**Table 2:** Primer sequences for RT-PCR.

Gene	Forward Primer	Reverse Primer
CD11b	CTGCTCCATCGCTGTCTG	TCTCCGTCTGGGACCTCA
CD11c	CAGGACCAGCAAGACCAC	GTTCAGCTCCACAGGCAC
MPO	CTGGACCTGCCTGCTCTGA	TGGGCGTGCCATACTGCT
MGAT3	GCCGCGTCATCAACGCCATCAA	CAGGTAGTCGTCGGCGATCCA
MGAT4A	GGCTATCACACCGATAGCTGGAG	TCCACCATTCCTTCTGCAACACC
MGAT4B	ACAACCCTCAGTCAGACAAGGAGG	GGTACCCTCAGAAGCCCGCAGCTT
MGAT5	GACCTGCAGTTCCTTCTTCG	CCATGGCAGAAGTCCTGTTT
FUT8	GACAGAACTGGTTCAGCGGAGA	GCAGTAGACCACATGATGGAGC
GAPDH	CGGAGTCAACGGATTTGGTCGTA	AGCCTTCTCCATGGTGGTGAAGAC

## Data Availability

The data used to support this study’s findings are available from the corresponding author upon reasonable request.

## Supplementary Materials

The following supporting information can be downloaded at: https://www.mdpi.com/article/10.3390/biom16050756/s1, Figure S1: Effects of ATRA-induced HL60 cells on expression levels of CD11b and β1,4-GlcNAc-branched N-glycans. Figure S2: Establishment of knockout cell lines of NB4 cells. Figure S3: Correlation gene analysis and comparison between healthy controls and APL patients in GSE13159. Figure S4: Original Western blot.

## Abbreviations

AML	Acute myeloid leukemia
APL	Acute promyelocytic leukemia
ATRA	All-trans retinoic acid
CHX	Cycloheximide
DSA	*Datura stramonium* agglutinin
E4-PHA	*Phaseolus vulgaris* erythroagglutinin
GEO	Gene Expression Omnibus
GLUT	Glucose transporter
GnT	N-acetylglucosaminyltransferase
KO	Knockout
L4-PHA	*Phaseolus vulgaris* leucoagglutinin
LCA	*Lens culinaris* agglutinin
MAPK	Mitogen-activated protein kinase
MPO	Myeloperoxidase
N/C	Nucleus/cytoplasm
WT	Wild-type
