# Supplementary material for: Upregulation of GnT-IVa and Its Critical Roles in ATRA-Induced Differentiation of Acute Promyelocytic Leukemia Cells
Source: Biomolecules. 2026 May 21;16(5):756. doi: 10.3390/biom16050756 (PMC13204627; doi:10.3390/biom16050756)
Supplement: Supplementary file 1 [file biomolecules-16-00756-s001.zip › biomolecules-4293074-Supplementary Figure S1-S3.pdf]

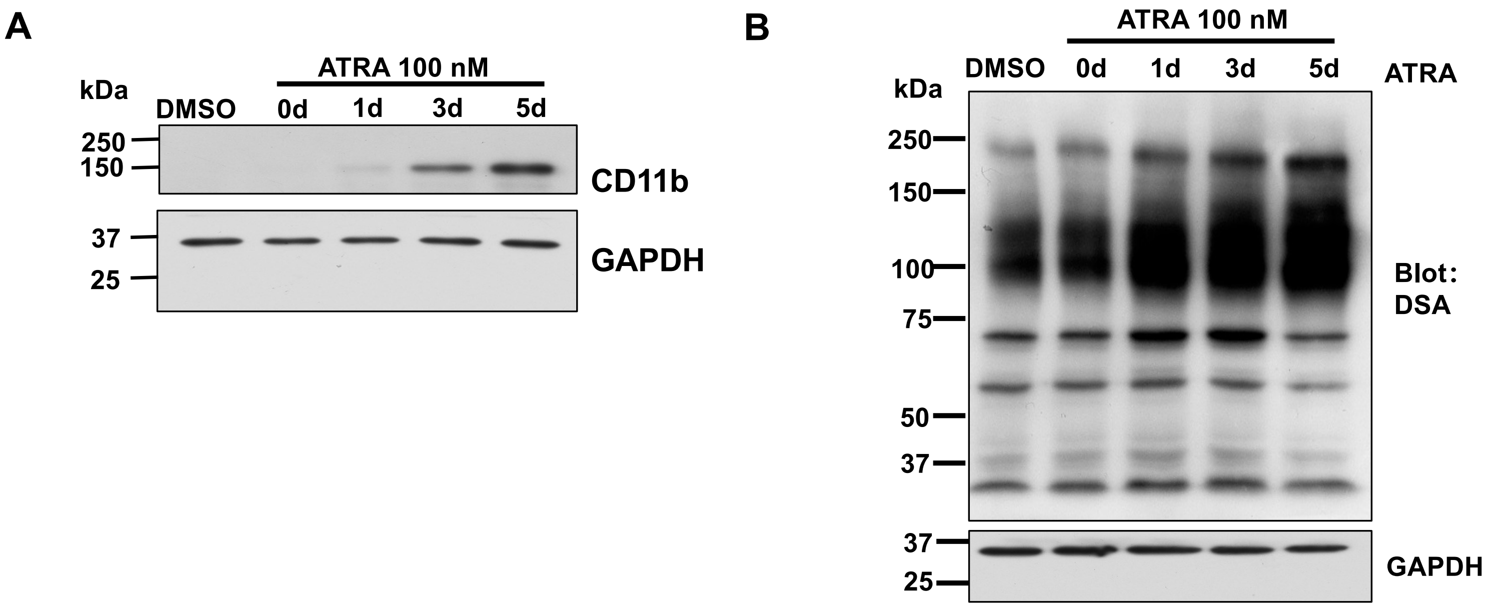

sFig.S1. Effects of ATRA-induced HL60 cells on expression levels of CD11b and β1,4-GlcNAc-branched N-glycans

(A) Western blots for CD11b expression in HL60 cells at the indicated time points, with GAPDH serving as a loading control.

(B). Equal amounts of protein from these cells were loaded onto a 7.5% SDS-PAGE gel to compare the expression levels of β1,4-GlcNAc-branched N-glycans using a lectin blot with DSA, with GAPDH serving as a loading control.

A

MGTA4A WT: TCATATCACCCCCTGAAAGCTATTATCCTGACTTGACAAACCT

KO-1 allele-1:   TCAT--CCTGACTTGACAAACCT

KO-1 allele-2:   TCATATCGACCCCTGAAAGCTATAAACCTGACTTGACTCCCCT

AAA

KO-2 allele-1:   TCATATCACCCCCCGAAAGCTATTATCCTGACTTGACAAACCT

KO-2 allele-2:   TCATATCACCCCATGATT-----------TATCCTGACTTGACAAACCT

B

MGAT4B WT: AGCCCGCAGGAGAAGGAGGACTCGGTCATCGTGGTGCTGA

KO-1 allele-1:   AGCCCGCAGGAGAAGGAGGACTCGGTC--TCGTGGTGCTGA

KO-1 allele-2:   TGACCGCACGAGAAAGAGGAGTCGGTCAATCGTGGTGCTGA

KO-2 allele-1:   AGCCCGCAGGAGAAGGATGA--------------------GTGGTTCAGA

KO-2 allele-2:   AGCCCGCAGGAGAAGGAGGACTCG--------------TGGTGCTGA

sFig.S2. Establishment of knockout cell lines of NB4 cells

(A) The MGAT4A-targeting gRNA was designed (underlined). Compared to WT cells, the sequence of MGAT4A KO-1 cells showed 22 bases deletion in allele-1 and 5 bases mutation (AAA inserted between A and A; two separate T to A substitutions at different positions) in allele-2, and T to C substitutions in allele-1 and 7 bases deletion; bases mutation (C to A substitutions) in allele-2 in the KO-2 NB4 cells.

(B) The MGAT4 B-targeting gRNA was designed (underlined). Compared to WT cells, the sequence of MGAT4B KO-1 cells showed a 1-base deletion in allele-1 and a 3-base mutation (A inserted between A and T; G to A substitution and C to G substitution) in allele-2, and a 10-base deletion in allele-1 and a 7-base deletion in allele-2 in the KO-2 NB4 cells.

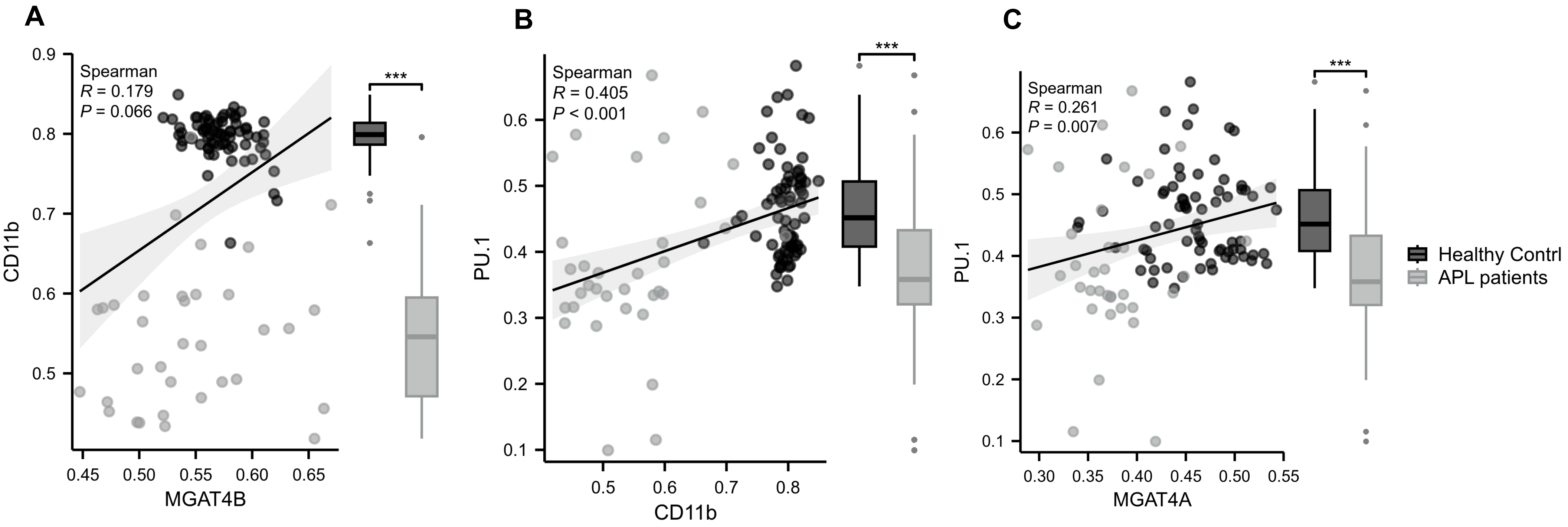

sFig.S3. Correlation gene analysis and comparison between healthy controls and APL patients in GSE13159 (A) Correlation between MGAT4B and CD11b expression. **(B)** Correlation between PU.1 and CD11b expression.**(C)** Correlation between PU.1 and MGAT4A expression.
