# Supplementary material for: Upregulation of GnT-IVa and Its Critical Roles in ATRA-Induced Differentiation of Acute Promyelocytic Leukemia Cells
Source: Biomolecules. 2026 May 21;16(5):756. doi: 10.3390/biom16050756 (PMC13204627; doi:10.3390/biom16050756)

Orginal WB Figures

FIG.1A

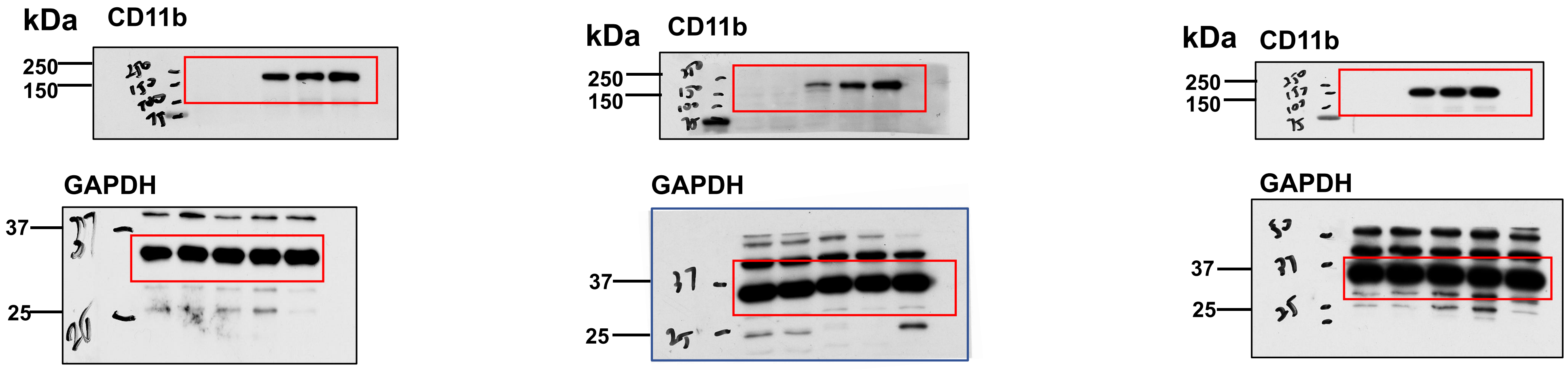

FIG.2A

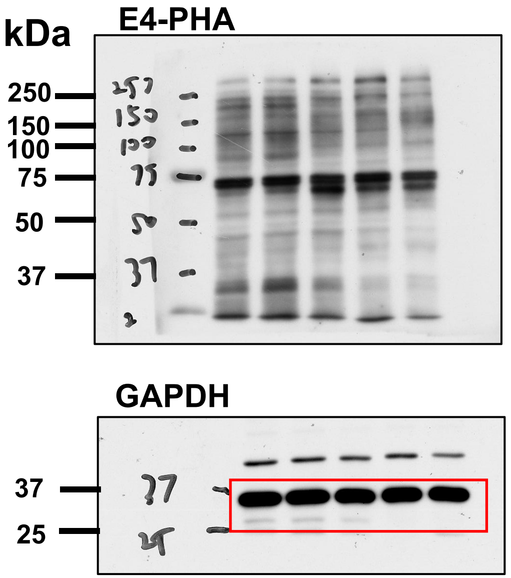

FIG.2B

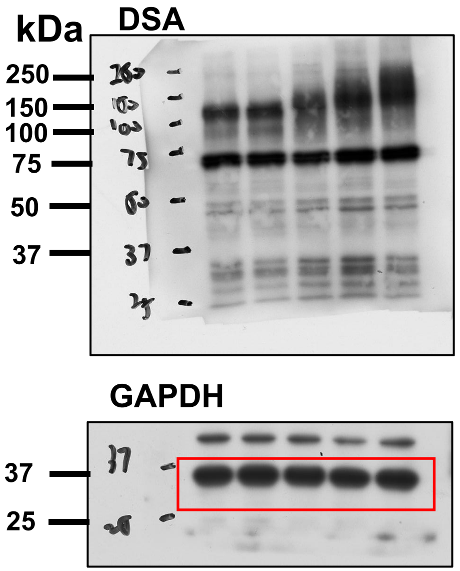

FIG.2C

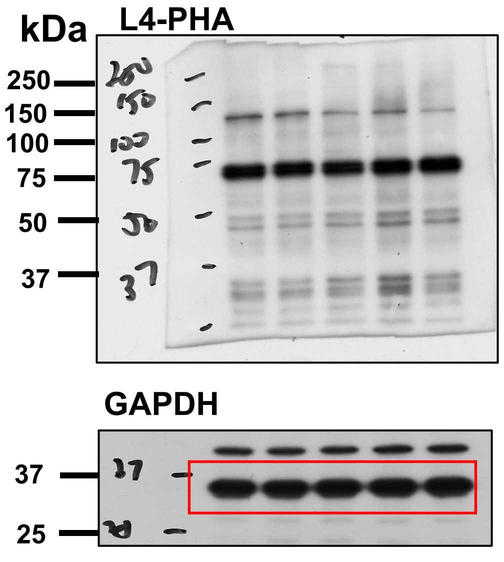

FIG.2D

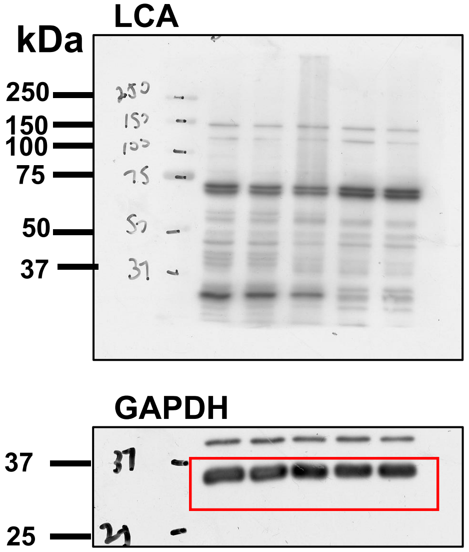

FIG.4A

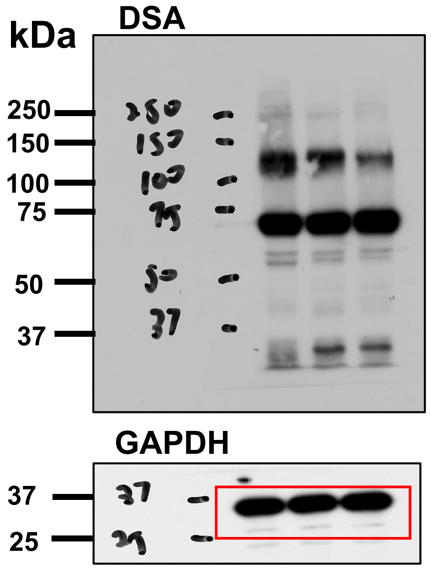

FIG. 4E

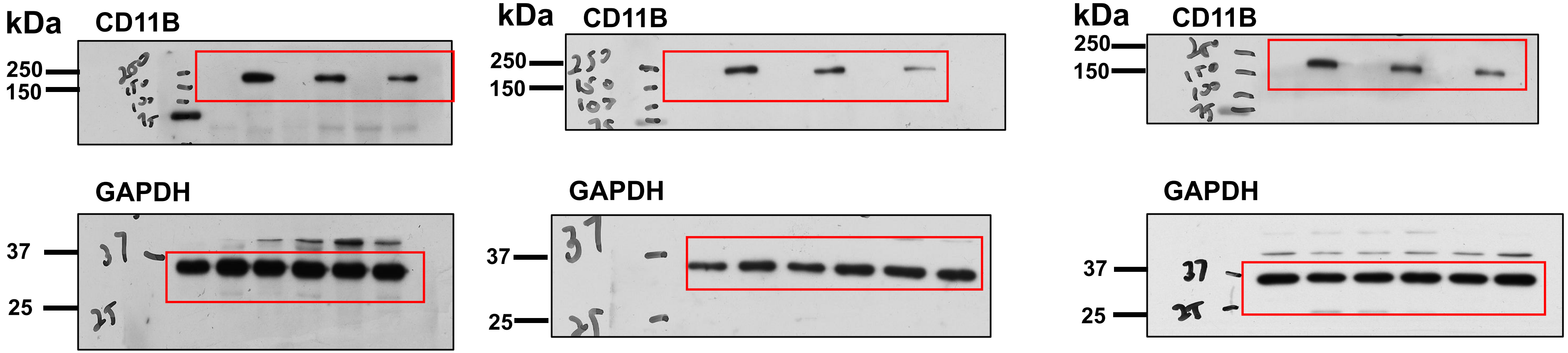

FIG. 5A

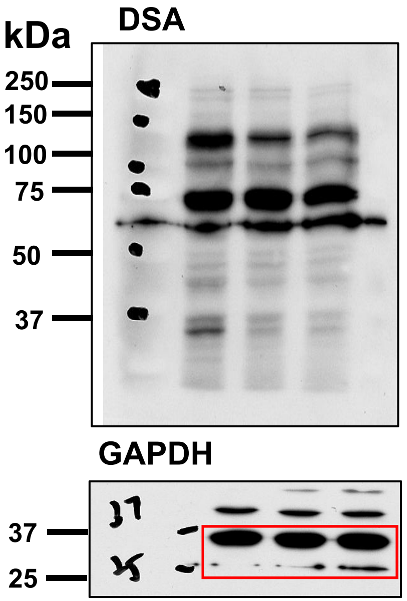

FIG. 5B

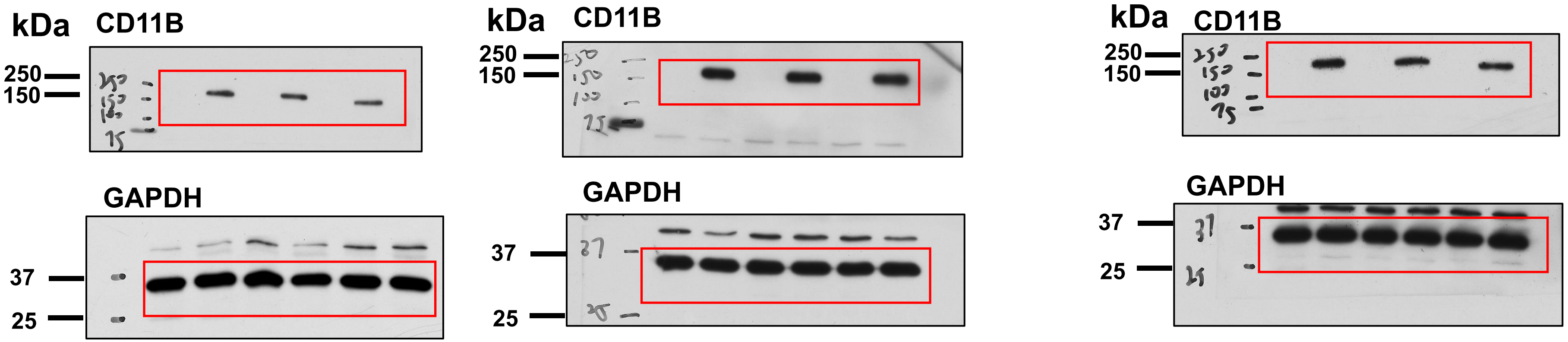

FIG.6A

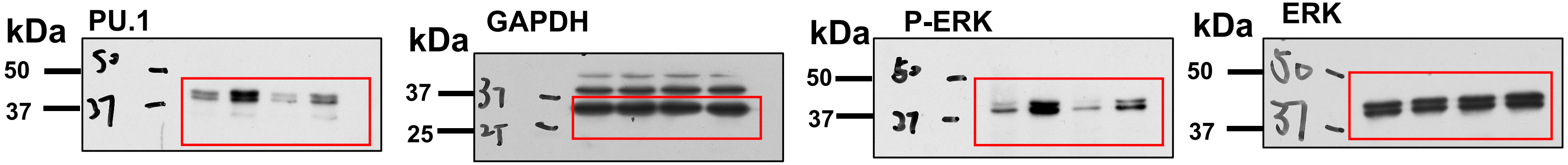

FIG.6B

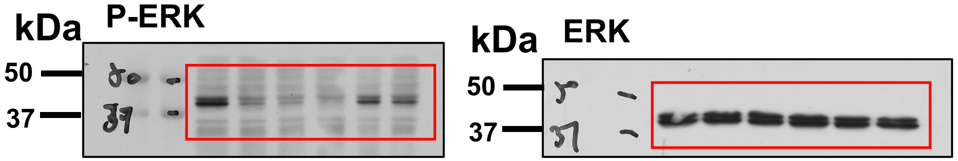

FIG.6D

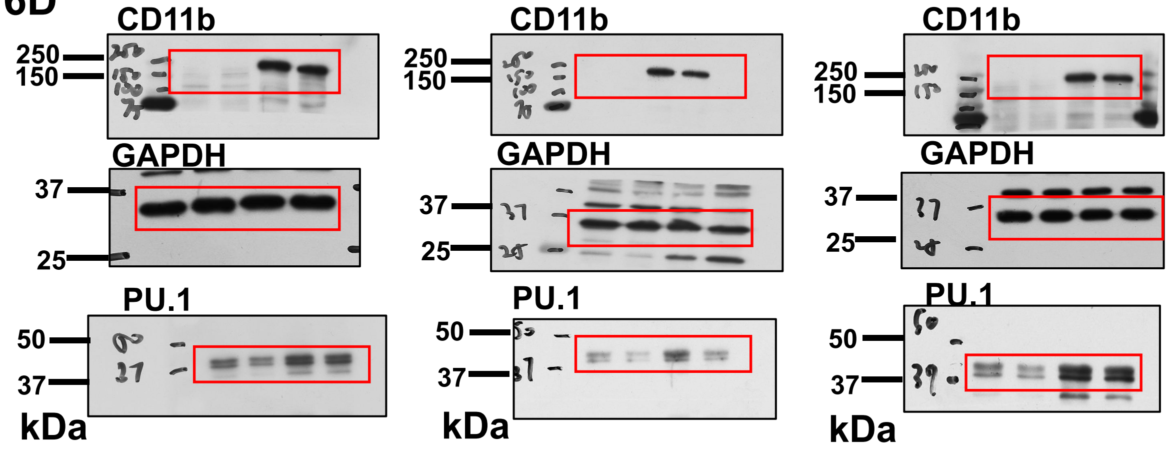

FIG.7A

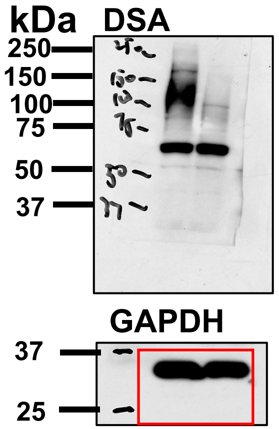

FIG.7B

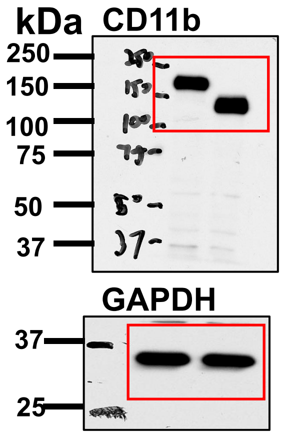

FIG.7D

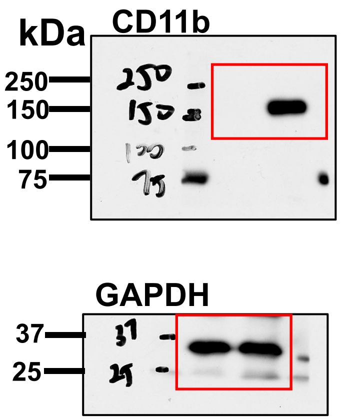

FIG.7E

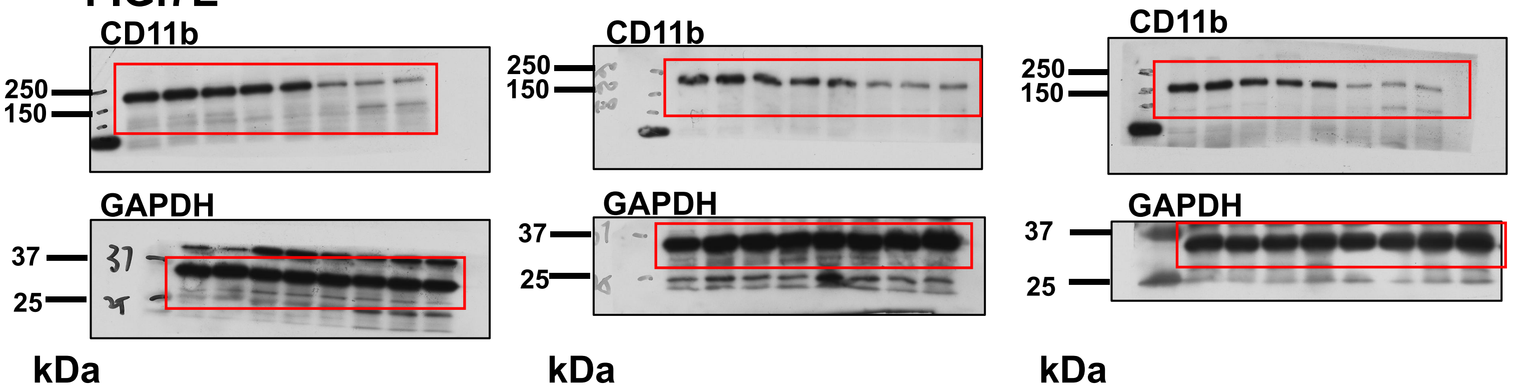

SFIG.1A

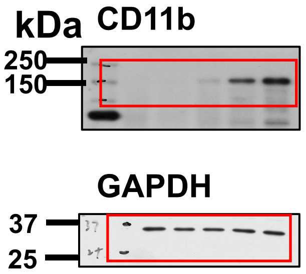

SFIG.1B

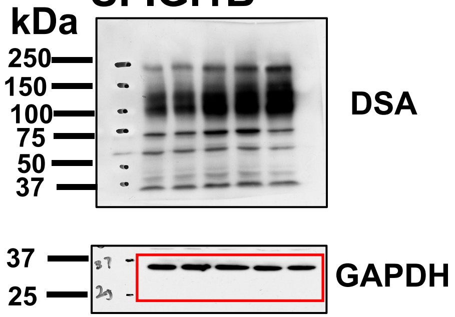

Supplement: Supplementary file 1 [file biomolecules-16-00756-s001.zip › biomolecules-4293074-Supplementary Figure S4.pdf]
